# Volumetric Changes of the Paranasal Sinuses with Age: A Systematic Review

**DOI:** 10.3390/jcm12103355

**Published:** 2023-05-09

**Authors:** Amaya Iturralde-Garrote, José Luis Sanz, Leopoldo Forner, María Melo, Clara Puig-Herreros

**Affiliations:** 1 Departament d’Estomatologia, Facultat de Medicina I Odontologia, Universitat de València, 46010 Valencia, Spain; 2 Clínica de Logopedia, Fundació Lluis Alcanyis, Universitat de València, 46010 Valencia, Spain

**Keywords:** paranasal sinuses, volumetric changes, sinus anatomy, age, systematic review, dentistry

## Abstract

Background: There are four pairs of paranasal sinuses: maxillary, ethmoidal, frontal, and sphenoidal. It is common to see changes in size and shape throughout life, so understanding the effect of age on sinus volume can help in radiographic studies and in planning dental and surgical procedures in the sinus–nasal region. The aim of the present systematic review was to perform a qualitative synthesis of available studies which assess the volumetric characteristics of the sinuses and their changes according to age. Materials and Methods: The present review followed PRISMA 2020 guidelines. A systematic advanced electronic search was performed in five databases (Medline (via PubMed), Scopus, Embase, Cochrane, and Lilacs) in June–July 2022. Studies that assessed the volumetric changes of paranasal sinuses with age were eligible for inclusion. A qualitative synthesis of the methodology and results of the included studies was performed. Quality assessment was performed using the NIH quality assessment tool. Results: A total of 38 studies were included in the qualitative synthesis. Most authors who studied the maxillary and ethmoidal sinuses concluded that it begins its development from birth until the maximum peak of growth, from which it begins to decrease in volume with age. Results regarding the volumetric changes of the frontal and sphenoidal sinuses are mixed. Conclusions: Based on the results of the studies included in the present review, it can be concluded that the volume of the maxillary and ethmoidal sinus appears to decrease with age. Conclusions on the volumetric changes of the sphenoidal and frontal sinuses require further evidence.

## 1. Introduction

There are four pairs of paranasal sinuses: maxillary, ethmoidal, frontal, and sphenoidal. They are spaces filled with air and lined with mucous membranes located in the maxillofacial region, centered in the skull, and communicating with the nasal cavity. They begin to develop from the primitive choana between 25 and 28 weeks of gestation. The sinuses, together with the nose, form a fundamental functional unit in the respiratory tract, in conjunction with the tracheal–bronchial tree and the lungs [1,2]. In addition, its presence contributes to the development and conformation of the craniofacial massif throughout human growth. Its size and shape vary from person to person and influence the size and shape of the face [3,4].

These structures are anatomically and physiologically related, forming a system with very specific functions, such as air conditioning, filtration, and warming of inspired air, as well as preparing an immune response to allergens, pollutants, and other particles to protect the lower airways [5]. In humans, the four paranasal sinuses are the most specialized structures for ventilation and are formed by the gradual pneumatization of solid bone tissue, a physiological process in which the sinus increases in volume and fills with air [6,7].

Another function attributed to the paranasal sinuses, specifically the sphenoid, maxillary, and frontal sinuses, is to act as a resonance chamber and transmission of nasal resonance; the state of these cavities (size and shape), especially the maxillary, directly influences the characteristics of the voice [3,8]. In this manner, small cavities reinforce the amplitude of the higher or acute frequencies, and large cavities reinforce the amplitude of the lower or grave frequencies. In addition, the vibration of sound through bone conduction can enhance the sensation of the acoustic resonances of the different sinuses [9,10].

The development and pneumatization of the sinuses continue after birth and undergo changes throughout life. The maxillary sinuses are the first to develop, are the largest in size, and are located in the cheeks. The roof of the sinus is formed by the floor of the orbit. It is a fragile wall that is crossed by the infraorbital nerve in its central part. The floor of the sinus is formed by the alveolar process of the maxilla and the hard palate. Its formation begins in the tenth week of intrauterine development, reaches a volume of approximately 6–8 cm^3^, and is filled with fluid until birth. In the postnatal period, there are two moments of rapid growth: a first period of transverse growth until the age of three years and a second of vertical growth between seven and twelve years, reaching the nostrils, the nasal-lacrimal duct, and the zygomatic recess. From there on, it continues its development slowly until it reaches its full maturation (volume) between twenty and thirty years of age [7,11].

The growth of the maxillary sinus is also induced by the eruption of the permanent dentition. The apices of the maxillary second premolars and first and second molars are in close relationship with the floor of the maxillary sinus. In some cases, only a thin layer of mucous membrane separates them, so a dental infection can easily spread into the sinus and cause acute sinusitis [5,12].

The sphenoid sinus is immersed in the thickness of the body of the sphenoid bone. It is asymmetrical and variable in shape and size. Its pneumatization begins around two years of age, progresses in the anteroposterior direction until the age of five, and reaches its full development between fifteen and thirty years of age. It is surrounded by neurovascular structures such as the pituitary gland; the internal carotid artery; and the optic, maxillary, and pterygoid nerves. During the pneumatization process, sinus expansion increases contact with these structures, which in many cases, protrude into the sinus [11,13].

The frontal sinuses are a pair of lobulated cavities located in the frontal bone behind the superciliary arches. They are not seen at birth, are the last to begin pneumatization, and are only detectable in radiological images between three and five years of age. Their development continues until approximately twenty years of age. Their shape is highly variable due to differences in pneumatization. It has been suggested that in patients older than sixty years, the frontal sinus has a greater volume due to bone resorption [12,14,15].

The ethmoidal sinus is located in the anterior area of the skull base, above and to the sides of the nasal cavity, and consists of a complex bony labyrinth of thin-walled cells (approximately eight to fifteen), which are divided into anterior and posterior according to their location in the ethmoid and their proximity to the middle turbinate. At birth, some cells are already present. The sinus completes its development around twelve years of age with a marked convexity of its lateral and medial walls. The pneumatization of this sinus is highly variable [5,16,17].

The ethmoidal and maxillary sinuses are pneumatized at birth, while the frontal and sphenoidal sinuses become pneumatized around the second and sixth years of life, respectively [18]. Generally, all pairs of sinuses are asymmetrical, and a correlation of their volume has been described, indicating a harmonic growth of all sinuses with each other. Genetic diseases, environmental conditions, infections, and aging can affect sinus structures. Therefore, it is common to see changes in size and shape throughout life, so understanding the effect of age on sinus volume can help in radiographic studies and in planning dental and surgical procedures in the sinus–nasal region [11,19,20].

However, no efforts have been made to review the available evidence on the volumetric change of the paranasal sinuses with age and reach conclusions in this regard. Therefore, the aim of the present systematic review was to perform a qualitative synthesis of available studies which assess the volumetric characteristics of the sinuses and their changes according to age.

## 2. Materials and Methods

The present study was carried out following the “Preferred Reporting Items for Systematic Reviews and Meta-Analyses” or PRISMA guidelines [21]. The register for our systematic review is https://doi.org/10.17605/OSF.IO/4U25K. Since this review was not on studies involving human participants, it was not eligible for register in PROSPERO. Instead, we opted for OSF Registries, as performed by previous similar studies.

This systematic review aims to establish current knowledge regarding volumetric changes in the paranasal sinuses (maxillary, sphenoid, ethmoid, and frontal) and possible age-related changes in people with no history of disease or surgery in the oro-rhino-sinus region. For this purpose, we posed the following question: Do the paranasal sinuses change their volume with age?

**Figure 1:**
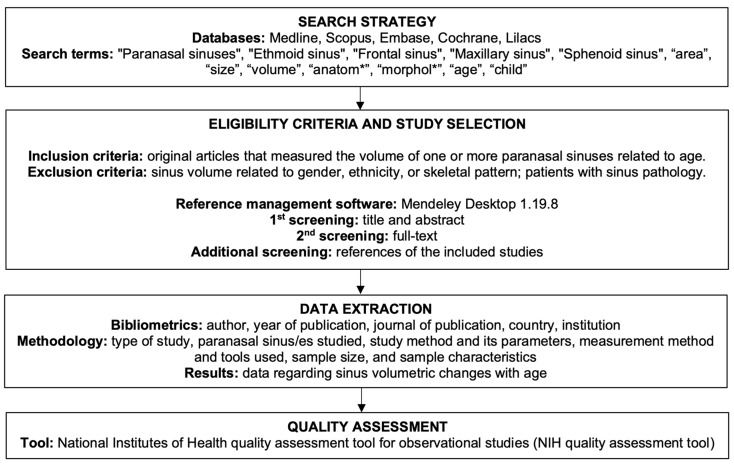
Systematic flowchart of the review methodology.

The question was elaborated following the PICOS framework [21]: population/problem (P): patients of any age without sinus pathology; intervention (I): measurement of sinus volume of any of the sinuses; comparison/control (C): different age groups; outcome (O): volumetric changes of the sinuses with age; study type (S): any. A systematic flow chart of the review methodology is presented in Figure 1.

### 2.1. Inclusion/Exclusion Criteria

Original articles that measured the volume of one or more paranasal sinuses and related it to the age of the patients were included in the study, regardless of the study method, and were eligible.

Studies that related the volume to gender, ethnic group, or skeletal pattern of the patient were excluded (unless they complied with the age information) and discarded, as well as those that studied such parameters in patients with sinus pathology.

### 2.2. Search Strategy

In order to identify the relevant studies for this systematic review, an electronic bibliographic search was carried out in the databases Medline (via PubMed), Scopus, Embase, Cochrane, and Lilacs. The search was conducted during the month of December 2022. The search and study selection process was conducted independently by two researchers (A.I.-G. and J.L.S.). In the event of doubt, a third researcher was consulted (L.F.).

Search terms: the search strategy included 5 Mesh (Medical Subject Heading) terms: “Paranasal sinuses”, “Ethmoid sinus”, “Frontal sinus”, “Maxillary sinus”, and “Sphenoid sinus”; 7 uncontrolled descriptors: “area”, “size”, “volume”, “anatom*”, “morphol*”, “age”, and “child*”. The Boolean operators OR, AND, and NOT were used to join search terms related to the research question.

The following search string was used: (((“frontal sinus” [Title/Abstract] OR “ethmoidal sinus” [Title/Abstract] OR “sphenoidal sinus” [Title/Abstract] OR “maxillary sinus” [Title/Abstract] OR “paranasal sinus” [Title/Abstract]) AND (area [Title/Abstract] OR size [Title/Abstract] OR volume [Title/Abstract] OR morphol* [Title/Abstract] OR anatom* [Title/Abstract])) AND (age [Title/Abstract])) NOT (child*).

### 2.3. Study Selection

The references yielded from the search strategy were exported to Mendeley Desktop v.1.19.8 (Elsevier Inc.; Amsterdam, The Netherlands). Duplicates were manually discarded using this software. After discarding duplicates, a first screening was performed based on article titles and abstracts of the articles, following the inclusion/exclusion criteria described previously. A second screening of the full text of the remaining studies was then performed to confirm their eligibility. Posteriorly, a screening of the references of the included studies was performed to look for additional potentially eligible studies.

### 2.4. Data Extraction

In order to perform the bibliometric analysis of the studies included in the review, the following data were collected: author, year of publication, journal of publication, country, and institution to which the authors were linked. For the qualitative synthesis of the methodology used by the included studies, the following variables were recorded: type of study, paranasal sinus/es studied, study method and its parameters, measurement method and tools used, sample size, and sample characteristics. For the results, the data provided by the articles regarding sinus volumetric changes with age were extracted. Statistical data were included for those studies where they were provided.

### 2.5. Quality Assessment

The quality assessment of the included studies was performed using the National Institutes of Health quality assessment tool for observational studies (NIH quality assessment tool; available at: https://www.nhlbi.nih.gov/health-topics/study-quality-assessment-tools, accessed on 10 January 2023).

## 3. Results

### 3.1. Study Selection and Flow Diagram

**Figure 2:**
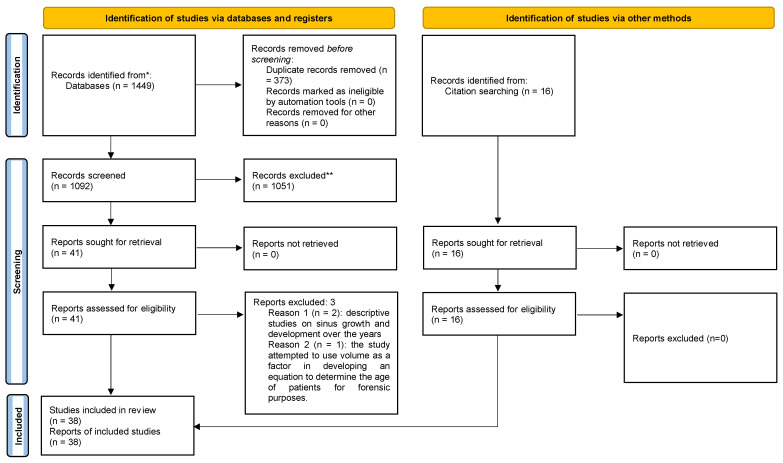
Systematic flow chart representing the study selection process. Based on the PRISMA 2020 flow diagram. * Only electronic databases were consulted. ** Records were excluded based on title and abstract screening.

The study selection process is illustrated in Figure 2. During the search, 1465 articles related to sinus morphometry were found. In the database search, 357 were found in PubMed, 726 in Embase, 276 in Scopus, and 90 in Lilacs. The search in the Cochrane database did not yield any results. Additionally, the screening of the references of the included studies yielded 16 new eligible articles. 

A total of 373 duplicates were excluded. From the remaining 1092 records, another 1051 were excluded after reading the title and abstract, as they did not meet the inclusion criteria. The remaining 41 articles were read in their entirety, and 3 additional studies were excluded: two of them because they were descriptive studies of sinus growth and development over the years and a third because it attempted to use volume as a factor in developing an equation to determine the age of patients for forensic purposes. Thus, 38 articles that met the inclusion criteria were included in the systematic review.).

### 3.2. Study Characteristics

All but two studies [22,23], which used cadaveric dissection techniques, were performed using imaging techniques. The techniques used were X-rays (3 studies), CBCT (8 studies), and tomography (25 studies). Table 1 details the various measurement methods used to determine the dimensions and volume of the paranasal sinuses in each article.

All articles included in the review were cross-sectional observational studies. Four studies were prospective [20,44,49,50], and the rest were retrospective.

**Figure 3:**
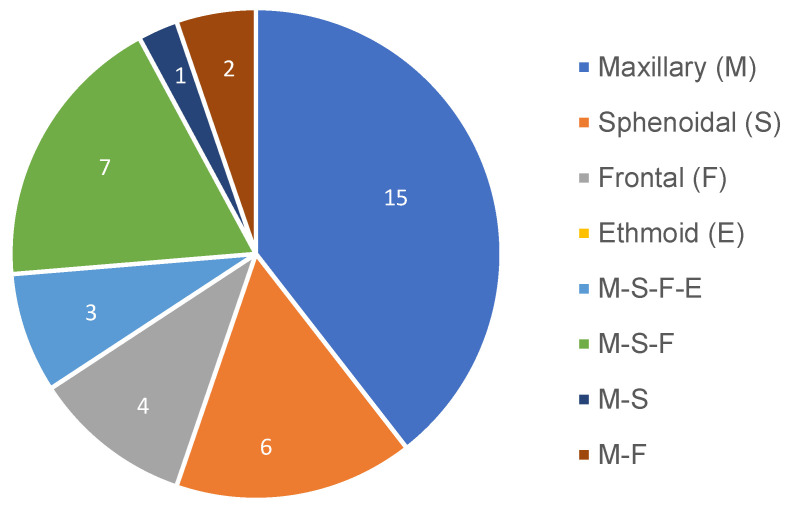
Pie chart representing the distribution of the studied paranasal sinuses among the included studies.

The most studied paranasal sinuses were the maxillary, frontal, and sphenoidal sinuses, either individually or in different combinations between them, which are detailed in Figure 3. Only three studies included the ethmoid sinuses [20,39,45].

The ages of the participants included ranged from 0 years to >100 years (Table 1). All studies were performed on living subjects, except those of Uchida et al., 1998 [22]; Takahashi et al., 2010 [40]; Demiralp et al., 2019 [33]; and Andrianakis et al., 2020 [23], which were performed on skulls.

**Figure 4:**
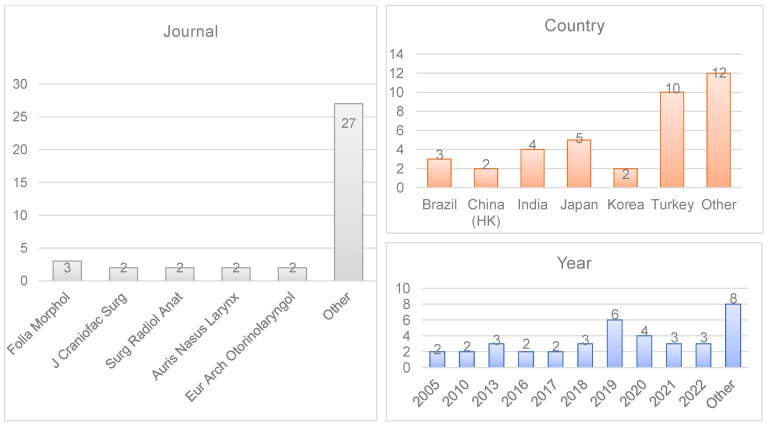
Bibliometric data of the included studies (year of publication, journal, and country).

All the authors of the included studies were linked to a university or health institution dedicated to education and research. The breakdown of the studies by year, country, and journal of publication is detailed in Figure 4.

### 3.3. Study Results

#### 3.3.1. Maxillary Sinus

Most authors who studied the maxillary sinus concluded that it begins its development from birth until the maximum peak of growth, from which it begins to decrease in volume with age [12,20,24,25,27,29,30,32,35,37,39,40,43,45,46,48,49,50]. In contrast, Jasso-Ramirez et al., 2022 [11], and de Barros et al., 2022 [28], concluded that maxillary sinus volume increases with age. In six of the studies, no relationship between sinus volume and age was found [6,7,22,31,33,36].

#### 3.3.2. Sphenoidal Sinus

Regarding the sphenoidal sinus, the authors Andrianakis et al., 2020 [23]; Kim et al., 2010 [6]; Demiralp et al., 2019 [33]; Oliveira et al., 2017 [13]; Singh et al., 2021 [38]; Takahashi et al., 2016 [40]; Yilmaz et al., 2020 [12]; and Özer et al., 2018 [47], did not find a linear correlation between sinus volume and age. In contrast, Kumar et al., 2022 [20]; Karakas and Kavakli, 2005 [27]; Cohen et al., 2018 [32]; Emirzeoglu et al., 2017 [39]; Ozdikici, 2018 [45]; Alasmari et al., 2019 [46]; and Yonetsu et al., 2000 [51], found that sinus volume decreases with age. Only Pirinc et al., 2019 [34], and Jasso-Ramirez et al., 2022 [11], reported an increase in sphenoid sinus volume.

#### 3.3.3. Frontal Sinus

A decrease in frontal sinus volume was observed in the studies of Kumar et al., 2022 [20]; Karakas and Kavakli, 2005 [27]; Tatlisumak et al., 2008 [15]; Emirzeoglu et al., 2007 [39]; and Ozdikici, 2018 [45]. In the studies of Jasso-Ramirez et al., 2022 [11]; Demiralp et al., 2019 [33]; Fatu et al., 2006 [42]; and Soman et al., 2016 [44], an increase in sinus area was observed, while Kim et al., 2010 [6]; Sahlstrand-Johnson et al., 2011 [31]; Cohen et al., 2018 [32]; Marino et al., 2020 [36]; Takahashi et al., 2016 [40]; Yilmaz et al., 2020 [12]; and Rubira-Bullen et al., 2010 [41], did not observe sinus volumetric alterations with age.

#### 3.3.4. Ethmoidal Sinus

The three studies that include the ethmoidal sinus in their measurements conclude that the sinus volume decreases with age after reaching its maximum growth peak [20,39,45].

The summary of the results of the articles is summarized in Table 2.

### 3.4. Quality Assessment

The quality analysis of all the articles was carried out using the NIH quality assessment tool. All the authors clearly defined both their study objective and the population (number, characteristics, and eligibility) on which they were carrying out the research (sections 1–3). Except for the studies performed on cadavers, all used similar populations, and their selection was carried out applying the same inclusion and exclusion criteria for all (section 4), but only two of the studies made a sample size calculation (section 5). The authors reported the exposure factors of the population before measuring the results, except for one of them (section 6). All investigators measured the varying degrees of exposure of the study population to the independent variables for a sufficient length of time to observe the results uniformly across the sample (sections 7–9). The participants in the studies were divided into groups according to age brackets so that those in charge of measuring the volumes knew the degree of exposure of each patient (section 11). Most studies took measures to control for confounding factors that could alter or mask the exposure–outcome relationship (section 14). Items 10, 12, and 13 were not considered applicable to the studies included in this literature review (Table 3).

## 4. Discussion

The four pairs of paranasal sinuses (maxillary, sphenoidal, frontal, and ethmoidal) are not only a fundamental part of the upper airway but also contribute to the development of the jaw and face and constitute the largest air cavity of the skull. The radiographic study of the paranasal sinuses is quite frequent because they are usually the site of chronic infectious processes, allergies, hypoplasia, pneumosinus dilatans, or atelectasis [39,45]. For example, sinusitis is one of the most common paranasal diseases, affecting almost 30 million people in the United States and 11% of the European population, making it one of the main causes of antibiotics prescription [41].

Since the paranasal sinuses are basically an air-filled space, one of the most important characteristics for their evaluation is the volume. Having well-defined parameters of the anatomy and normal evolution of the sinus complex is of great help to clinicians and surgeons in its diagnosis and treatment. At present, with increasing life expectancy, knowing the volumetric changes that sinuses undergo with age is an important reference for planning clinical procedures [11,20,52,53]. Accordingly, the aim of this study was to perform a systematic review of the change in volume experienced by the paranasal sinuses throughout life.

In all articles, we found imaging as the method of study. The only exceptions were Uchida et al., 1998 [22], and Andrianakis et al., 2020 [23], who made silicone models of the sphenoid sinuses from cadaver heads, which they then used to measure the volume. Four techniques were used within imaging methods: computed tomography (CT) [6,11,12,13,15,19,20,24,25,26,27,30,31,32,34,36,39,40,43,45,47,48,49,50,51], cone beam computed tomography (CBCT) [7,28,29,33,35,37,38,46], chemically developed radiographs [41,44], and digitized chemically developed radiographs [42].

Most studies used digital methods for image analysis, using specific automatic or semi-automatic study software [6,7,11,12,13,15,19,20,24,25,26,27,28,29,30,31,32,33,34,35,36,37,38,40,42,43,46,47,48,49,50,51]. Other authors made use of non-digital methods on paper media [39,41,44,45].

The Cavalieri Principle is a stereological method of volume measurement. It allows measuring a three-dimensional characteristic, such as volume, from two-dimensional images. These images should be consecutive slices of the organ to be studied, on which a template of points are superimposed to measure the area and, subsequently, the volume [45,54].

This makes it a good technique to be used in radiographic studies performed with CT or CBCT. In addition, the technique has proven to be accurate, fast, reliable, and possible to apply, even without a computer program, since it can be performed on paper prints of the images [20,39]. This was the method used to determine sinus volume by Elamin et al., 2021 [25]; Kumar et al., 2022 [20]; Karakas and Kavakli, 2005 [27]; Emirzeoglu et al., 2007 [39]; Ozdikici, 2018 [45]; and Jasim and Al-Taei, 2013 [50].

Other stereological methods used to calculate sinus volume were the ellipsoid formula [12,24,31,44,48], other formulas [30,46], and the measurement of sinus dimensions [15,41,49].

Another group of studies employed volume measurement tools integrated into the image analysis software [6,7,11,13,19,26,28,29,32,33,34,35,36,37,38,40,42,43,47,51].

Finally, Uchida et al., 1998 [22], and Andrianakis et al., 2020 [23], used the liquid displacement method (based on Archimedes’ Principle) to determine the volume of the sphenoidal sinuses by introducing the silicone models that they had taken out of the skulls included in their study, into a calibrated cylinder filled with water. This is an appropriate method for studying the volume of irregular structures, but it requires that the sample population are cadavers.

The most used study methods were diagnostic imaging, including three-dimensional imaging. This type of radiological technique, which can recognize very slight changes in tissue density, allows for the contour and characteristics of the bony structures and cavities of the skull to be observed in more detail [55]. 

Their images are accurate and reproducible, they are non-invasive methods, and the development of three-dimensional analysis software facilitates the measurement of the morphometric characteristics of the structures with greater accuracy than the stereological methods used in two-dimensional studies. Although CT was used in a greater number of studies, it has some disadvantages compared to CBCT, such as higher cost, higher radiation dose, and longer exposure time that can cause artifacts and worse image quality due to patient movements [53,56,57].

By far, the most studied sinus of all was the maxillary sinus. It appears in 14 articles individually and, in 13, studied in combination with other sinuses. Possibly this is because, in addition to being the largest paranasal sinus, its location in the maxilla and the close relationship it has with the orbit, nasal cavity, and dental apices make it an anatomical structure of interest for procedures such as sinus endoscopy, maxillofacial surgery, and dental surgery (implants and sinus lift) [19,25,58]. It is also widely studied in the forensic field for the identification of individuals because its structure remains intact, even in cases of death by cremation [7]. 

In 20 of the articles studying the maxillary sinus, the authors conclude that its volume decreases with age [12,19,20,24,25,26,27,29,30,32,35,37,39,40,43,45,46,48,49,50]. However, in five studies, no relation is found between sinus volume and age [6,7,31,33,36]. This difference may be attributed to the characteristics of the sample population and the ages included in the sample. In the majority of articles where an increase in volume is observed, individuals over 70 years of age are included in the sample, and in those that do not observe changes, only participants up to 65 years of age are included. 

The growth of the maxillary sinus remains stable until the sixth decade of life. After the seventh decade, its volume begins to decrease due to a loss of minerals in the bone matrix (osteoporosis) that surrounds the sinus, which shrinks its walls and reduces its volume. Loss of volume has also been reported in the sinuses of totally or partially edentulous persons. Both factors occur in older people [5,25,26]. This may explain why the investigations of Jasso-Ramirez et al., 2022 [11], and de Barros et al., 2022 [28], whose sample consisted of young individuals, obtained an increase in sinus volume.

In contrast, the ethmoidal sinus was the least studied (only three articles), perhaps because the complexity of its anatomy makes it too difficult to perform an accurate and standardized measurement of its anatomy. Moreover, some consider that from an embryological and anatomical point of view, the frontal sinus and ethmoidal cells should be considered as a single structure due to their closeness [32,42].

In the sphenoid sinus study, Jasso-Ramirez et al., 2022 [11], and Pirinc et al., 2019 [34], found that the volume increased. Their study populations were almost entirely young patients under 60 years of age. The outcome of the remaining studies was divided between those that measured a decrease in volume [20,27,32,39,45,46,51] and those that did not find a volume–age correlation [6,12,13,23,33,38,40,47]. 

When comparing the characteristics of the studies of each group, we see that they differ by the measurement methods; in the volume decrease group, all (except Cohen et al., 2018 [32]) use stereological measurement methods, and in the group that found no relationship, they employ digital methods by using computer software (except Andrianakis et al., 2020 [23]). 

Since the sphenoid sinus is an irregularly shaped and difficult-to-access cavity enclosed within the body of the sphenoid and is considered the most variable cavity in the human body, it is possible that non-three-dimensional measurement methods may affect the results. Because of its proximity to vital neurovascular and endocrine structures, the sphenoid sinus is of great clinical relevance, and knowledge of its anatomy and volume is indispensable in planning skull base surgeries [6,11,13,23,32,38].

When analyzing the results obtained in frontal sinus investigations, we see similar results to those of the sphenoid sinus. For Kumar et al., 2022 [20]; Karakas and Kavakli, 2005 [27]; Tatlisumak et al., 2008 [15]; Emirzeoglu et al., 2007 [39]; and Ozdikici, 2018 [45], sinus volume decreases with age. All use stereological measurement methods. In contrast, Kim et al., 2010 [6]; Sahlstrand-Johnson et al., 2011 [31]; Cohen et al., 2018 [32]; Marino et al., 2020 [36]; Takahashi et al., 2016 [40]; Yilmaz et al., 2020 [12], and Rubira-Bullen et al., 2010 [41], found no relationship between sinus volume and age. All used digital study methods, except Rubira-Bullen et al., 2010 [41]. 

Only Jasso-Ramirez et al., 2022 [11]; Demiralp et al., 2019 [33]; Fatu et al., 2006 [42]; and Soman et al., 2016 [44], detected an increase in sinus volume. It has been described that in elderly patients, the thinning of the bony cortex of the sinus due to osteoporosis can increase sinus volume and thin the cortex of the orbit. Nevertheless, its size is highly variable and can be affected by genetic, environmental, and pathologic factors, craniofacial morphology, and even hormonal levels [42,44]. For example, a recent study reported that cystic fibrosis patients were more prone to present frontal sinus aplasia and hypoplasia [59].

Only three authors made measurements of ethmoid sinus volume: Kumar et al., 2022 [20]; Emirzeoglu et al., 2007 [39]; and Ozdikici, 2018 [45]. All three saw a decrease in sinus volume, used similar study and measurement methods, and populations in the same age ranges.

Despite the heterogeneity of the studies in study methods, measurement, and population, when performing the quality analysis, we observed that all studies followed a similar structure, with a sufficient introduction stating the objectives of the study, appropriate description of the methodology and population used, as well as an explanation of the results and statistical methods. Despite this, only two articles measured the necessary sample size, and because of the type of studies reviewed, neither were the groups randomized nor the investigators blinded.

The methodological heterogeneity in terms of the characteristics of the sample and the parameters used for the measurement of sinus volume made it impossible to perform a quantitative synthesis or meta-analysis. The use of standardized methodology and previously established age groups could be useful in the future to reach statistically significant conclusions [60]. Instead, a qualitative synthesis of the available evidence was presented to provide a global view of the evidence in this regard, with the inherent limitations that this may imply.

The volumetric investigation of the paranasal sinuses is not only useful to know the amount of air that these structures can contain. Studying the sinus volume allows for learning its anatomy, pneumatization, and degenerative and pathological processes. Knowing the normal dimensions and volumes of the sinuses at each stage of life allows for recognizing sinus alterations or deformities and planning endoscopic surgeries at the base of the skull without putting adjacent structures, facial reconstruction surgeries, or dental implants at risk. Knowing normality allows for the recognition of abnormality [11,23,32,58]. 

To the best of our knowledge, the possible impact of sinus volumetric change on voice resonance, and its implication in presbyphonia, has not been described and may be an interesting topic for future studies.

The substantial heterogeneity of study and measurement methodologies hinders the comparison between studies. Future studies should implement a standardized three-dimensional sinus volume measurement method based on new diagnostic imaging technologies and 3D reconstruction software.

## 5. Conclusions

Based on the results of the included studies, the ethmoidal and maxillary sinuses experience development and growth from 0 to 20 years, reaching their maximum peak of development. From 20 to 50 years, a discrete decrease in volume may be observed. From 50 to 65 years of age, the decrease in volume is accentuated, which is further accelerated from 65 years onward. Conclusions on the volumetric changes of the sphenoidal and frontal sinuses require further evidence.

These results are based on heterogeneous methodologies and samples with varying characteristics. Therefore, they should be interpreted as an approximation and not as an exact measure. 

## Figures and Tables

**Table 1:** General characteristics of the study methodology.

Author, Year and Country	Studied Sinus	Equipment Used and Parameters	Methods Used to Assess Sinus Volume	Sample Characteristics
Andrianakis et al., 2020 [23], Austria	Sphenoidal	Cadaveric dissection. Models of hydrophilic addition silicone sinuses.	The models were immersed in a 0.5 cm^3^ graduated cylinder filled with water.	50 skulls (100 sinuses); 65–100 years
Samhitha et al., 2019 [24], India	Maxillary	CT scans	Software Radianat (the following formula was used: width × ant-post. × height. × 0.52)	100 participants; 1–90 years
Elamin et al., 2021 [25], Sudán	Maxillary	CT scans (Toshiba Aquilion 64 CT scanner); 120 kVp; 210 mA; 1 s rotation; 64 × 0.625 collimator; 220 mm FOV	Software Image J; Cavalieri Principle: by the number of pixels of each slice, the area was calculated (1–6 mm slices), and the volume was calculated with the formula: Vmax = t × ∑A	81 participants (46 M 35 W); 17–78 years
Kim et al., 2010 [6], Republic of Korea	Frontal; Sphenoidal Maxillary	CT scans; 1 mm sections; 140 kVp; 120 mA	Software Vworks 4.0 (Automatic volume calculation during 3D reconstruction)	60 participants (46 M 14 W); 18–63 years
Jasso-Ramírez et al., 2022 [11], Mexico	Frontal; Sphenoidal; Maxillary	CT scans (Light Speed plus CT, GE medical services); 0.625 collimator; 1.25 mm sections; 50 mA; 120 kVp	Multiplanar reconstruction with Centricity Universal Viewer software	210 scans (104 M 106 W); 0–20 years
Kumar et al., 2022 [20], India	Frontal; Maxillary; Ethmoid; Sphenoidal	CT scans (Bright Speed Elite 16, Wipro, GE)	The tomography slice area was calculated, and by multiplying it by the slice thickness, the volume was calculated. By adding all the partial volumes together, the total volume was calculated according to the Cavalieri Principle	300 participants (163 M 137 W); 17–65 years
Sarilita et al., 2021 [19], Indonesia	Maxillary	CT scans (SOMATOM Definition DS dual source 128, Siemens)	Semi-automatic volume calculation with software ITK-SNAP 3.0	194 sinuses; 0–25 years
Jun et al., 2005 [26], Republic of Korea	Maxillary	CT scans; 120 kVp; 180 mA; 2.5 mm sections	The volume was measured in 3D reconstruction of the sinus with Vworks software.	238 sinuses/175 participants (109 M 109 W); 0–80 years
Karakas y Kavakli, 2005 [27], Turkey	Maxillary; Sphenoidal; Frontal	CT scan (Prospeed helical CT, GE); 120 kVp; 160 mA	The tomography slice area was calculated, and by multiplying it by the slice thickness, the volume was calculated. By adding all the partial volumes together, the total volume was calculated according to the Cavalieri Principle	91 participants (47 M 44 W); 5–55 years
de Barros et al., 2022 [28], Brazil	Maxillary	CBCT (iCAT); 23 × 17 cm; 40 s; 0.3 mm voxel, 0.5 mm focus	The volume was measured in 3D reconstruction of the sinus with the software DDS-Pro 2.14.2-2022	161 participants (72 M 89 W); 6–18 years
Bornstein et al., 2019 [29], Hong Kong, China	Maxillary	CBCT (ProMax 3D Mid, Planmeca); 0.2–0.4 mm voxel; 10 × 6–8 × 8–10 × 10–20 × 6–20 × 10–20 × 17 FOV	Romexis v.4.4.0.R software	87 CBCT/174 sinuses (27 M 60 W); 18–82 years
Ariji et al., 1994 [30], Japan	Maxillary	CT scans (SOMATOM DR scanner, Siemens); 125 kVp; 350 mA; 2–4 mm sections	Cosmozone ISA (Nikon) image analysis software. The volume was calculated with the formula: $V\;=\;\sum_{i=1}^{n}si.ti.\cos d$	115 participants (58 M 57 W): 4–94 years
Sahlstrand-Johnson et al., 2011 [31], Sweden	Maxillary; Frontal	CT scans (SOMATOM Sensation 16, Siemens)	Volume was measured twice: 1. Software Leonardo WorkStation (Siemens) in an automatic way; 2. Using the formula: width × ant-post × skull-flow diameter × 0.5	60 participants (28 M32 W); 18–65 years
Cohen et al., 2018 [32], Israel	Maxillary; Sphenoidal; Frontal	CT scans	Automatic measurement with volume tracing in advanced vessel analysis software (Philips)	201 participants (201 M 100 W); 25–>65 years
Demiralp et al., 2019 [33], Turkey	Maxillary; Frontal; Sphenoidal	CBCT (ProMAx 3D Max, Planmeca)	3D reconstruction and measurement with Invivo 5.1.2 software (Anatomage Inc.)	32 skulls (18 M 14 W) 41.4 ± 10.2–39.6 ± 9.2 years
Gulec et al., 2020 [7], Turkey	Maxillary	CBCT (3D Accuitomo 170, Morita J); 90 kVp; 5 mA; 17.5 s exp.; 17 × 12 cm area	3D reconstruction and measurement with MIMICS 21.0 software	133 participants/266 sinuses (49 M 84 W); 8–51 years
Pirinc et al., 2019 [34], Turkey	Sphenoidal	CT scan (SOMATOM Flash, Siemens); 120 kVp; 160 mA; 0.5 s exp.; 64 × 0.625 collimator; 220 mm FOV	Automatic measurement with Snygo Via software (Siemens)	200 participants (99 M 101 W); 4–84 years
Belgin et al., 2019 [35], Turkey	Maxillary	CBCT (i.CAT vision system, Imaging Sciences Intl.); 120 kVp; 5 mA; 8–9 s exp.; 0.3 mm isotropic voxel; 16 × 13 image area	The volume was calculated with the 3D tool of the MIMICS 19.0 software.	200 participants (86 M 114 W); 18–>65 years
Marino et al., 2020 [36], USA	Maxillary; Frontal	CT scans	3D volumetric analysis and APPS score	323 scans >13 years
Velasco-Torres et al., 2017 [37], Spain	Maxillary	CBCT (i-CAT Next Generation, Imaging Sciences Intl.); 120 kVp; 5 mA; 16 × 8 cm FOV; 10.8 s exp.; 0.3 mm voxel	ViewForum software 3D volume measurement tool (Philips Healthcare)	394 participants (193 M 201 W); 10–87 years
Tatlisumak et al., 2008 [15], Turkey	Frontal	CT scans (Emotion Tomography Machine, Siemens)	Measurement of sinus lengths with a DICOM image viewer	300 participants (123 M 177 W); 20–83 years
Oliveira et al., 2017 [13], Brazil	Sphenoidal	CT scans (SOMATOM AR star, Siemens); 110 kVp; 83 mA	Automatic volume measurement with the ITK/SNAP software	47 scans (20 M 27 M); 18–86 years
Singh et al., 2021 [38], Hong Kong, China	Esf enoidal	CBCT (Promax 3D Mid, Planmeca); 90 kVp 0.4 mm voxel, 9.4 s exp. 20 × 17 cm FOV	Software Romexis v.4.4.0.R (Planmeca)	148 participants (285 sinuses) (71 M 77 W); 15–85 years
Emirzeoglu et al., 2007 [39], Turkey	Frontal; Maxillary; Sphenoidal; Ethmoid	CT scans (Cytec 3000i, GE); 120 kVp; 130 mA; 3 mm sections	Printed images + template to measure densities (square grid test); Cavalieri Principle. The following formula was used: $V=t\times \left[\frac{\left(SU\right)\times d}{SL}\right]\times \sum P$	77 participants (39 M 38 W); 18–72 years
Takahashi et al., 2016 [40], Japan	Maxillary; Frontal; Sphenoidal	CT scan (Asteion super 4, Toshiba); 120 kVp; 225 mA; 1 mm sections	3D reconstruction of volumes with Osirix v.6.0.2 software (Pixmeo)	77 skulls (33 M 44 W); <69–>100 years
Yilmaz et al., 2020 [12], Turkey	Maxillary; Frontal; Sphenoidal	CT scan (Alexion, Toshiba); 120 kVp; 120 mA; 3 mm sections	Images were analyzed with Osirix MD v.8.0 software (Pixmeo) and volume was measured with the ellipsoidal volume formula: π/6 × transverse diameter × ant-post diameter × cranial-caudal diameter.	47 participants (26 M 21 W); 65–81 years 47 participants (21 M 26 W); 20–49 years
Rubira-Bullen et al., 2010 [41], Brazil	Frontal	Radiographs (Caldwell projection)	Measurement on X-rays (negatoscope) with ruler	145 participants (116 M 29 W); 17–>51 years
Fatu et al., 2006 [42], Romania	Frontal	Digital radiographs	Volume was measured automatically with Imageberger v.4.02 software.	60 participants; 4–83 years
Iwai et al., 1995 [43], Japan	Maxillary	CT scans (TCT-700 S, Toshiba), 2 mm sections	Volume was measured with Area Curve Meter X-PLAN 360 software (Ushikata).	70 participants (36 M 34 W); 17–87 years
Soman et al., 2016 [44], India	Frontal	Cephalogram X-ray; 70–75 kVp; 8 mA	The sinus contour was traced on special paper and measured in cm; the magnification factor (height and width) was subtracted.	200 participants (100 M 100 W)
Özdikici, 2018 [45], Turkey	Frontal; Maxillary; Ethmoid; Sphenoidal	CT scans; 3–5 mm sections	Cavalieri Method. Template ”square grid test” for calculating area and then volume. The following formula was used: $V=\sum_{}^{}Pi.ap.t$	125 participants (68 M 57 W); 18–75 years
Alasmari et al., 2019 [46], Saudi Arabia	Maxillary; Sphenoidal	CBCT (Galileo, Sirona); 12 × 15 × 15 cm^3^ FOV; 85 kVp; 5–7 mA; 2 s exp.	The volume of the sinuses was calculated with the formula for the volume of the sphere (V_1_ = 4/3 πr3) and the volume of the pyramid (V_2_ = 1/3 A × h)	50 participants; 21–80 years
Özer et al., 2018 [47], Turkey	Sphenoidal	CT scans (Activion 16 CT scanner, Toshiba); 120 kVp; 100 mA 2 mm sections, 240 FOV	Osirix (Pixmeo) software and ROI volume program	144 participants (89 M 55 W); 9–83 years
Abdulhameed et al., 2013 [48], Nigeria	Maxillary	CT scans (Neusoft Dual slide helical CT); 15 cm FOV; 120 kVp; 200 mA 5 mm sections	3D reconstruction with V-works 3.0 software. Volume was calculated with the product of cranial-caudal, ant-post, and transverse diameters and slice thickness.	130 participants (79 M 51 W); 20–80 years
Baweja et al., 2013 [49], India	Maxillary	CT scans (Helicoidal CT/e spiral CT, GE); 125 kVp; 80–160 mA; 5 mm sections	The ant-post, transverse, and vertical dimensions were measured.	90 participants; 0–>61 years
Jasim y Al-Taei, 2013 [50], Irak	Maxillary	CT scans (Aquilion 64, Toshiba)	The volume of the sinus was calculated by sections and then the total with the formula: $V=\sum_{\iota =1}^{\eta }dS\times \Delta h$	120 participants (60 M 60 W); 40–69 years
Yonetsu et al., 2000 [51], Japan	Sphenoidal	CT scans (Helicoidal HiSpeed Advantage SG CT, GE); 1–3 mm collimator; 23 cm FOV; 512 × 512 matrix; 120 kVp; 100 mA	3D reconstruction of the sinuses	161 participants (85 M 76 W); 1–80 years
Uchida et al., 1998 [22], Japan	Maxillary	Cadaveric dissection Models of hydrophilic addition silicone sinuses	The models were immersed in a graduated cylinder filled with water	32 skulls (59 sinuses) (20 M (36 sinuses) 12 W (23 sinuses)); 46–94 years

M: men; W: women.

**Table 2:** Study results.

Author, Year and Country	Studied Sinus	Results/Obtained Measurements
Andrianakis et al., 2020 [23], Austria	Sphenoidal	No linear correlation was found between age and sinus volume. Spearman’s correlation analysis showed no significant correlation between the two parameters (*p* = 0.707).
Samhitha et al., 2019 [24], India	Maxillary	Sinus volume does decrease with age. In men, the volume reaches its maximum value between 41 and 50 years, and in women between 51 and 60 years; from that age, it gradually decreases.
Elamin et al., 2021 [25], Sudan	Maxillary	A negative correlation was found between sinus volume and age, r = 0.029 (*p* = 0.9) in the male group and r = 0.313 (*p* = 0.07) in the female group. Sinus volume decreases with age. This decrease is more pronounced in women.
Kim et al., 2010 [6], Republic of Korea	Frontal; Sphenoidal Maxillary	No changes in sinus volume with age were found.
Jasso-Ramírez et al., 2022 [11], Mexico	Frontal Sphenoidal Maxillary	Statistically significant differences were found between the different age groups. The volume of sinuses increases with age and reaches its maximum at 15 years of age.
Kumar et al., 2022 [20], India	Frontal Maxillary Ethmoid Sphenoidal	Sinus volume has an inverse correlation with age. From the age group 17–26 years, the volume of the sinuses gradually decreases as the patient’s age increases. Considering the average volume values obtained for the right-sided sinuses in the extreme age groups, they summarized: Frontal: 17–26 years (15.11 + 4.95 cm^3^) 47–55 years (10.67 + 4.39 cm^3^) *p* = 0.826. Maxillary: 17–26 years (31.97 + 8.97 cm^3^) 47–55 years (21.14 + 8.74 cm^3^) *p* = 0.047 Ethmoid: 17–26 years (9.68 + 2.62 cm^3^) 47–55 years (6.00 + 3.02 cm^3^) *p* = 0.377 Sphenoidal: 17–26 years (9.39 + 5.53 cm^3^) 47–55 years (6.33 + 3.14 cm^3^) *p* = 0.052
Sarilita et al., 2021 [19], Indonesia	Maxillary	The average sinus volume increases from the first year of age until the 16–20 years group, after which it begins to decrease. Group 0–5 years (1361.12 mm^3^); group 16–20 years (13,278.73 mm^3^); group 20–25 years (12,325.21 mm^3^).
Jun et al., 2005 [26], Republic of Korea	Maxillary	Sinus development continues until the 30s in men (maximum volume 24.043 mm^3^) and 20s in women (maximum volume 15,859.5 mm^3^). From that age, the volume decreases.
Karakas y Kavakli, 2005 [27], Turkey	Maxillary Sphenoidal Frontal	The volume of sinuses increases, in men, until the age group 21–25 years (maxillary—31.97 ± 8.97 cm^3^; frontal—8.83 ± 4.46 cm^3^; sphenoidal—9.68 ± 2.62 cm^3^); in women, it increases until the age group 16–20 years (maxillary—21.81 ± 7.83 cm^3^; frontal—3.51 ± 3.11 cm^3^; sphenoidal—8.71 ± 2.44 cm^3^). After these age groups, the volume decreases.
de Barros et al., 2022 [28], Brazil	Maxillary	Sinus size increases with age. The mean values of sinus volume were lower in the group aged 6–11 years (8.560,61 mm^3^) than in those aged 12–17 years (10,678.83 mm^3^) and >18 years (12,329.65 mm^3^).
Bornstein et al., 2019 [29], Hong Kong, China	Maxillary	Subjects in the 18–24.3 years group had a larger sinus volume (17.16 cm^3^) than those in the 24.4–82 years group (14.72 cm^3^). Sinus volume decreases with age.
Ariji et al., 1994 [30], Japan	Maxillary	Sinus volume increased until the age of 20 years, with a correlation coefficient of 0.72. After that, it decreased with a correlation coefficient of −0.43.
Sahlstrand-Johnson et al., 2011 [31], Sweden	Maxillary Frontal	No correlation was found between sinus volume and age.
Cohen et al., 2018 [32], Israel	Maxillary Sphenoidal Frontal	Maxillary and Sphenoidal sinus volumes decrease with age. This is evidenced by a negative Pearson correlation coefficient with age (−0.34 and −0.24, respectively). No volume changes with age were found for the frontal sinus.
Demiralp et al., 2019 [33], Turkey	Maxillary Frontal Sphenoidal	Frontal sinus volume increases statistically with age. Sphenoidal and maxillary sinuses are not affected.
Gulec et al., 2020 [7], Turkey	Maxillary	No correlation was found between sinus volume and age.
Pirinc et al., 2019 [34], Turkey	Sphenoidal	Sinus volume increases until the age of nine years. At ten years, it reaches its size, which remains stable throughout life.
Belgin et al., 2019 [35], Turkey	Maxillary	Sinus volume decreased with age. It was significantly larger in the 18–24 years group (33.59 cm^3^) than in the >55 years group (25.23 cm^3^).
Marino et al., 2020 [36], USA	Maxillary Frontal	No correlation was found between breast volume and age.
Velasco-Torres et al., 2017 [37], Spain	Maxillary	Sinus size does decrease with age (rho = −0.249 and −0.186, and *p* < 0.001, right and left, respectively).
Tatlisumak et al., 2008 [15], Turkey	Frontal	Sinus size does decrease with age. Especially after 60 years of age.
Oliveira et al., 2017 [13], Brazil	Sphenoidal	According to Spearman’s correlation analysis, no linear correlation was found between breast volume and age.
Singh et al., 2021 [38], Hong Kong, China	Sphenoidal	No relationship was found between breast volume and age.
Emirzeoglu et al., 2007 [39], Turkey	Frontal Maxillary Sphenoidal Ethmoid	A negative correlation was found between the total volume of the 4 paranasal sinuses and age (r = −0.238; *p* < 0.05). This means that the total volume of the sinuses tends to decrease with age.
Takahashi et al., 2016 [40], Japan	Maxillary Frontal Sphenoidal	The maxillary sinus decreases with age after 90 years of age. No relationship between volume and age was found for the frontal and sphenoidal sinuses.
Yilmaz et al., 2020 [12], Turkey	Maxillary Frontal Sphenoidal	Maxillary sinus volume decreased significantly in the elderly participants (18.31 mm^3^ right/18.45 mm^3^ left) compared to the young participants’ group (22.2 mm^3^ right/23.11 mm^3^ left). No significant differences were observed for frontal and sphenoidal sinuses.
Rubira-Bullen et al., 2010 [41], Brazil	Frontal	There are no significant differences in sinus size between the different age groups and no change with age.
Fatu et al., 2006 [42], Romania	Frontal	The sinus begins to develop from the age of 4 years, reaches its adult size at about 16 years, and remains stable until about 60 years of age, when it increases in size, possibly due to bone resorption.
Iwai et al., 1995 [43], Japan	Maxillary	The sinus area increases and reaches its maximum growth peak between the third and fifth decade of life. After the age of 50 years, it begins to decrease.
Soman et al., 2016 [44], India	Frontal	The frontal sinus area increases with age. In men, this value decreases after 45 years, while in women, it continues to increase with age.
Özdikici, 2018 [45], Turkey	Frontal Maxillary Ethmoid Sphenoidal	The volume of the sinuses is highest at 20 years of age and then gradually decreases. There is a negative correlation between age and the total volume of the four paranasal sinuses (r = −0.240; *p* < 0.05).
Alasmari et al., 2019 [46], Saudi Arabia	Maxillary Sphenoidal	Sinus volume increases with age (21–30 years: 10.0 cm^3^ Maxillary/7.9 cm^3^ Sphenoidal), and there is a uniform pattern of growth until 51–60 years (16.1 cm^3^ Maxillary/9.0 cm^3^ Sphenoidal). Thereafter the volume decreases as aging progresses (71–80 years: 2.7 cm^3^ Maxillary/1.1 cm^3^ Sphenoidal).
Özer et al., 2018 [47], Turkey	Sphenoidal	There is no linear correlation between sinus volume and age.
Abdulhameed et al., 2013 [48], Nigeria	Maxillary	Maxillary sinus volume decreases after the age of 20 years. Although there is no uniform pattern of volume decrease with age, a decrease was found on both the right (from 14.15 cm^3^ to 13.51 cm^3^) and left side (from 14.33 cm^3^ to 9.61 cm^3^) when comparing the extreme age groups (20–29 years and 80–89 years, respectively).
Baweja et al., 2013 [49], India	Maxillary	Sinus size and volume increase until the second decade of life and thereafter decrease. Up to the age of 20 years, the changes are anteroposterior, and in adults, the greatest decrease is in the vertical direction.
Jasim y Al-Taei, 2013 [50], Irak	Maxillary	Sinus volume decreases with age.
Yonetsu et al., 2000 [51], Japan	Sphenoidal	The volume of the sinus increases until the third decade of life, after which it decreases. The mean maximum volume was 8.2 + 0.5 cm^3^ and decreased to 71% in the seventh decade.
Uchida et al., 1998 [22], Japan	Maxillary	No statistically significant differences were found in breast volume measurements between the different age groups.

**Table 3:** Quality assessment.

Author, Year and Country	Items													
	1	2	3	4	5	6	7	8	9	10	11	12	13	14
Andrianakis et al., 2020 [23], Austria	Y	Y	Y	N	N	Y	Y	Y	Y	NA	Y	NA	NA	Y
Samhitha et al., 2019 [24], India	Y	Y	Y	Y	N	Y	Y	Y	Y	NA	Y	NA	NA	N
Elamin et al., 2021 [25], Sudán	Y	Y	Y	Y	N	Y	Y	Y	Y	NA	Y	NA	NA	Y
Kim et al., 2010 [6], Republic of Korea	Y	Y	Y	Y	N	Y	Y	Y	Y	NA	Y	NA	NA	N
Jasso-Ramírez et al., 2022 [11], México	Y	Y	Y	Y	N	Y	Y	Y	Y	NA	Y	NA	NA	Y
Kumar et al., 2022 [20], India	Y	Y	Y	Y	N	Y	Y	Y	Y	NA	Y	NA	NA	Y
Sarilita et al., 2021 [19], Indonesia	Y	Y	Y	Y	N	Y	Y	Y	Y	NA	Y	NA	NA	Y
Jun et al., 2005 [26], Republic of Korea	Y	Y	Y	Y	N	Y	Y	Y	Y	NA	Y	NA	NA	Y
Karakas y Kavakli, 2005 [27], Turquía	Y	Y	Y	Y	N	Y	Y	Y	Y	NA	Y	NA	NA	Y
de Barros et al., 2022 [28], Brasil	Y	Y	Y	Y	N	Y	Y	Y	Y	NA	Y	NA	NA	Y
Bornstein et al., 2019 [29], Hong Kong, China	Y	Y	Y	Y	N	Y	Y	Y	Y	NA	Y	NA	NA	Y
Ariji et al., 1994 [30], Japón	Y	Y	Y	Y	N	Y	Y	Y	Y	NA	Y	NA	NA	Y
Sahlstrand-Johnson et al., 2011 [31], Suecia	Y	Y	Y	Y	N	Y	Y	Y	Y	NA	Y	NA	NA	Y
Cohen et al., 2018 [32], Israel	Y	Y	Y	Y	N	Y	Y	Y	Y	NA	Y	NA	NA	Y
Demiralp et al., 2019 [33], Turquía	Y	Y	Y	N	N	Y	Y	Y	Y	NA	Y	NA	NA	Y
Gulec et al., 2020 [7], Turquía	Y	Y	Y	Y	Y	N	Y	Y	Y	NA	Y	Y	NA	Y
Pirinc et al., 2019 [34], Turquía	Y	Y	Y	Y	N	Y	Y	Y	Y	NA	Y	NA	NA	Y
Belgin et al., 2019 [35], Turquía	Y	Y	Y	Y	N	Y	Y	Y	Y	NA	Y	NA	NA	Y
Marino et al., 2020 [36], E.E.U.U.	Y	Y	Y	Y	N	Y	Y	Y	Y	NA	Y	NA	NA	Y
Velasco-Torres et al., 2017 [37], España	Y	Y	Y	Y	N	Y	Y	Y	Y	NA	Y	NA	NA	Y
Tatlisumak et al., 2008 [15], Turquía	Y	Y	Y	Y	N	Y	Y	Y	Y	NA	Y	NA	NA	Y
Oliveira et al., 2017 [13], Brasil	Y	Y	Y	Y	N	Y	Y	Y	Y	NA	Y	NA	NA	Y
Singh et al., 2021 [38], Hong Kong, China	Y	Y	Y	Y	Y	Y	Y	Y	Y	NA	Y	NA	NA	Y
Emirzeoglu et al., 2007 [39], Turquía	Y	Y	Y	Y	N	Y	Y	Y	Y	NA	Y	NA	NA	Y
Takahashi et al., 2016 [40], Japón	Y	Y	Y	N	N	Y	Y	Y	Y	NA	Y	NA	NA	Y
Yilmaz et al., 2020 [12], Turquía	Y	Y	Y	Y	N	Y	Y	Y	Y	NA	Y	NA	NA	Y
Rubira-Bullen et al., 2010 [41], Brasil	Y	Y	Y	Y	N	Y	Y	Y	Y	NA	Y	NA	NA	N
Fatu et al., 2006 [42], Rumanía	Y	Y	Y	Y	N	Y	Y	Y	Y	NA	Y	NA	NA	N
Iwai et al., 1995 [43], Japón	Y	Y	Y	Y	N	Y	Y	Y	Y	NA	Y	NA	NA	N
Soman et al., 2016 [44], India	Y	Y	Y	Y	N	Y	Y	Y	Y	NA	Y	NA	NA	Y
Özdikici 2018 [45], Turquía	Y	Y	Y	Y	N	Y	Y	Y	Y	NA	Y	NA	NA	Y
Alasmari et al., 2019 [46], Arabia Saudí	Y	Y	Y	Y	N	Y	Y	Y	Y	NA	Y	NA	NA	N
Özer et al., 2018 [47], Turquía	Y	Y	Y	Y	N	Y	Y	Y	Y	NA	Y	NA	NA	Y
Abdulhameed et al., 2013 [48], (Nigeria)	Y	Y	Y	Y	N	Y	Y	Y	Y	NA	Y	NA	NA	Y
Baweja et al., 2013 [49], India	Y	Y	Y	Y	N	Y	Y	Y	Y	NA	Y	NA	NA	N
Jasim y Al-Taei, 2013 [50], Irak	Y	Y	Y	Y	N	Y	Y	Y	Y	NA	Y	NA	NA	N
Yonetsu et al., 2000 [51], Japón	Y	Y	Y	Y	N	Y	Y	Y	Y	NA	Y	NA	NA	Y
Uchida et al., 1998 [22], Japón	Y	Y	Y	N	N	Y	Y	Y	Y	NA	Y	NA	NA	Y

1. Study objective; 2. study population; 3. eligibility rate; 4. inclusion/exclusion; 5. sample size calculation; 6. measurement of exposure factors; 7. follow-up time; 8. measurement of exposure levels; 9. definition of exposure factors; 10. exposure measurement frequency; 11. results measurement; 12. blinding; 13. follow-up, 14; management of confounding factors. Y: yes; N: no; NA: non-applicable.

## Data Availability

The data presented in this study are available on request from the corresponding author.
